# Surgical outcomes of Bethesda System for Reporting Thyroid Cytopathology diagnostic category class I, II, and III thyroid nodules

**DOI:** 10.3389/fendo.2026.1822111

**Published:** 2026-05-25

**Authors:** Sang-Wook Park, Ga Young Lee, Min Ji Kim, Minsu Kwon, Young Ho Jung, Seung-Ho Choi, Dong Eun Song, Yoon Se Lee

**Affiliations:** 1 Department of Otolaryngology–Head and Neck Surgery, Gyeongsang National University Hospital, Jinju, Republic of Korea; 2 Department of Otolaryngology-Head and Neck Surgery, Asan Medical Center, University of Ulsan, College of Medicine, Seoul, Republic of Korea; 3 Department of Pathology, Asan Medical Center, University, University of Ulsan, College of Medicine, Seoul, Republic of Korea

**Keywords:** atypia, Bethesda classification, sonography, thyroid carcinoma, thyroid nodule

## Abstract

**Objectives:**

According to the Bethesda System for Reporting Thyroid Cytopathology, surgery is recommended for Bethesda classes I–III thyroid nodules only in selected situations, such as large or growing tumors with compressive symptoms or clinical suspicion of malignancy. Clinical findings and cytopathologic evaluation are often considered together to reduce discrepancies between preoperative cytology and final pathology. This study aimed to evaluate surgical outcomes in Bethesda classes I–III nodules and to identify risk factors predictive of malignancy.

**Methods:**

In this retrospective review, we included patients who underwent thyroidectomy for thyroid nodules classified as Bethesda classes I–III based on preoperative US-guided fine-needle aspiration (FNA) between 2010 and 2020. We collected data including preoperative cytopathology and ultrasound findings to analyze the risk factors related to malignancy and to evaluate treatment outcomes.

**Results:**

Among 192 patients, carcinoma was confirmed in 62 (32.3%). The malignancy rates for Bethesda classes I, II, and III were 23.5%, 20.4%, and 49.4%, respectively. The malignant histologic types included papillary thyroid carcinoma (PTC; *n* = 34, 54.8%) and follicular thyroid carcinoma (FTC; *n* = 23, 37.1%), while five cases (8.1%) showed coexisting histologic types, including multiple PTC variants or a combination of minimally invasive FTC and PTC. Bethesda class III showed a significantly higher incidence of malignancy than the other classes (*p* < 0.001). In a subgroup analysis of class III nodules, larger nodule size on ultrasonography and atypia with both nuclear and architectural features (AUS-N/A) were associated with malignancy (*p* = 0.045 and *p* = 0.028, respectively). In a multivariate analysis, AUS-N/A was an independent predictor of malignancy (odds ratio 13.275, 95% confidence interval 1.354–130.3, *p* = 0.026).

**Conclusion:**

Bethesda class III nodules showed a higher risk of malignancy. Surgical intervention should be considered, particularly when AUS-N/A is identified in Bethesda class III nodules. Even in cases where carcinoma was confirmed after surgery for Bethesda classes I, II, and III, the probability of recurrence was very low and the prognosis is favorable.

## Introduction

Thyroid nodules are a highly prevalent condition worldwide, and advancements in ultrasonography (USG) have contributed to an increased diagnostic rate of malignancy. Fine-needle aspiration (FNA) or core needle biopsy (CNB) of thyroid nodules has proven to be a safe and accurate procedure, significantly improving the diagnosis of malignancy (1–3). The Bethesda System for Reporting Thyroid Cytopathology (TBSRTC, Bethesda class) has provided a standardized six-tiered classification system for FNA specimens, which has played a pivotal role in improving communication between physicians and in reducing unnecessary thyroidectomies (4).

According to the practice guidelines for thyroid nodules such as the American Thyroid Association (ATA) guideline for thyroid nodule, surgical resection is not routinely recommended for non-diagnostic or unclear thyroid nodules classified as Bethesda classes I, II, or III after USG-guided FNA or CNB (5, 6). However, exceptions are made in cases where the tumor is large, demonstrates growth with compressive symptoms, raises clinical concerns for malignancy, or yields positive results on molecular testing (5, 6). In such instances, thyroidectomy may be considered, and malignancy is occasionally identified in the final pathological report after surgery. To decrease the rate of the discrepancy between preoperative cytopathologic reports gained from USG-guided FNA and postoperative pathologic finding, additional diagnostic tools including repeated FNA, CNB, molecular testing, and elastography have been applied to improve the diagnostic accuracy for non-diagnostic thyroid nodules. CNB yields larger tissue samples, allowing for better histological assessment. Although CNB presented a better diagnostic rate (70.78%) than repeated FNA (35.8%), a substantial number of CNB results were also inconclusive (7).

Next-generation sequencing (NGS) panel combined with cytology has significantly enhanced the sensitivity for detecting malignancy, especially in nodules that would otherwise yield non-diagnostic or indeterminate results. This approach to identify mutations such as *BRAF^V600E^*, *RAS*, and *TERT* promoter mutations improves the sensitivity of detecting malignancies from 72% (cytology alone) to 90%, which is a variable according to the Bethesda categories (8–10). Some commercially available molecular tests such as Afirma GSC, ThyroSeq GC, and ThyGeNEXT + ThyraMIR have been used for indeterminate cytology (8–10).

The choice of molecular test depends on the institutional or national prevalence of thyroid cancer and cytology practices. The cost-effectiveness of these tests remains a topic of ongoing evaluation, but they can reduce unnecessary diagnostic surgeries by accurately stratifying the risk of malignancy in low-risk nodules that do not require immediate intervention. These advanced diagnostic tools are not widely used yet. Thus, other risk stratification strategies based on preoperative TBSRTC system and clinical findings will help in deciding the treatment modality, either diagnostic lobectomy or active surveillance.

This study aims to analyze the specific risk factors for malignancy by collecting cases within Bethesda categories I, II, and III that proceeded to surgery. By comparing preoperative USG findings and FNA with final postoperative histopathology, this study was performed to reveal the malignancy risks in a specific finding based on the Bethesda reporting system.

## Patients and methods

### Patient selection

We conducted a retrospective review of patients who underwent thyroidectomy for thyroid nodules classified as Bethesda categories I, II, or III based on preoperative ultrasound-guided fine-needle aspiration (FNA) between January 2010 and December 2020.

The inclusion criteria were as follows:

1. Patients with Bethesda I–III thyroid nodules who underwent surgical resection.
2. Availability of preoperative ultrasonographic and cytopathologic data.
3. Presence of at least one clinical indication for surgery, including:
  - Nodule growth, defined according to the 2015 American Thyroid Association criteria as a >20% increase in at least two dimensions, >2 mm increase in any dimension, or >50% increase in volume.
  - Large nodule size (≥4 cm).
  - Compressive symptoms (e.g., globus sensation, dysphagia, or dyspnea).
  - Tracheal deviation confirmed on chest X-ray or computed tomography (CT).
  - Suspicious or indeterminate cytologic findings on repeated FNA.
  - Patient preference, including cosmetic concerns or anxiety regarding malignancy.

The exclusion criteria included the following:

1. Patients younger than 18 years or older than 80 years.
2. Patients with incomplete clinical, cytologic, or imaging data.
3. Patients without clearly documented indications for surgery.

A total of 224 patients were initially identified, and 32 were excluded due to incomplete records or insufficient clinical information, resulting in 192 patients included in the final analysis. This study was approved by the Institutional Review Board of Asan Medical Center (IRB 2025-0209).

### Treatment principle

Upon detection of a thyroid nodule, the USG impression of the thyroid nodule was classified according to the Korean Thyroid Imaging Reporting and Data System (K-TIRADS), with irregular margin, punctate echogenic foci, and taller-than-wide shape defined as malignant features (1). An increase in nodule size was defined based on the 2015 ATA criteria: greater than 20% increase in two dimensions, an increase of more than 2 mm in any dimension, or greater than 50% increase in total volume (5).

Tracheal deviation was defined as the displacement of the trachea caused by the thyroid tumor, as observed on chest X-ray or neck CT, by comparing it to an imaginary line that connects the hyoid bone to the suprasternal notch. Compressive symptoms were defined as the presence of globus sensation, dysphagia, or dyspnea at the time of thyroid nodule diagnosis.

Cytology was evaluated according to the 2023 TBSRTC. In cases of Bethesda class III, the nodules were further categorized into “nuclear” atypia and “others” atypia group. Nuclear atypia was defined as nuclear enlargement, nuclear grooves, or irregular nuclear membranes. “Others” atypia includes architectural atypia, oncocytic atypia, atypical lymphoid cells, and not-otherwise specified (NOS) atypia. Architectural atypia was defined as three-dimensional clustering of follicular cells or a coiled arrangement. Specifically, we classified cases with “both nuclear and architectural” atypia (N/A atypia), while all others were categorized as “others atypia”. Both N/A atypia was defined as nodules showing the features of both nuclear and architectural atypia together (4).

### Statistical analysis

The variables were defined based on the USG features of the nodules, cytopathologic characteristics of FNA results, and the presence or absence of clinical symptoms. To compare differences between benign and malignant groups, *T*-tests, chi-square tests, and Fisher’s exact tests were performed. For continuous variables, the data were presented as medians with corresponding ranges. Significant factors from the univariate analysis were then included in a multivariate analysis to identify independent predictors of malignancy. All statistical analyses were conducted using SPSS version 21, with a *p*-value <0.05 considered statistically significant.

## Result

**Figure 1:**
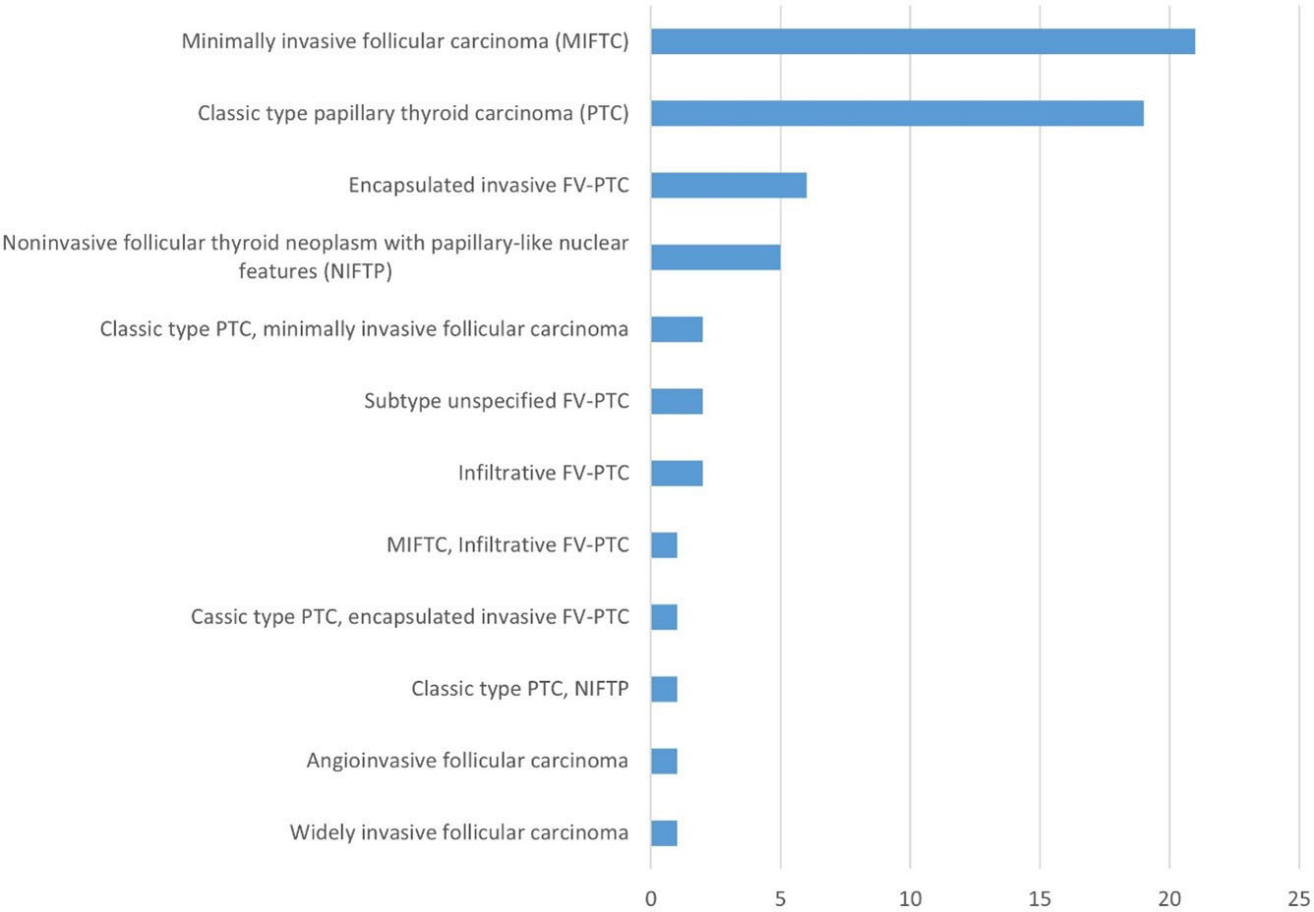
Distribution of pathological diagnoses in patients who underwent thyroid surgery. Minimally invasive follicular carcinoma and classic-type papillary thyroid carcinoma were the most common subtypes, followed by encapsulated invasive FV-PTC and NIFTP.

The number of patients who underwent surgery of Bethesda classes I, II, and III nodules was 192, consisting of 51 men and 141 women. The distribution of Bethesda classes I, II, and III nodules was 17, 98, and 77, respectively, with 130 benign cases and 62 malignant cases. The mean age of the benign group was 50.7 ± 14.5 years, while that of the malignant group was 54.8 ± 12.1 years. The malignant histologic types included papillary thyroid carcinoma (PTC; *n* = 34, 54.8%) and follicular thyroid carcinoma (FTC; *n* = 23, 37.1%). In addition, five cases (8.1%) showed coexisting histologic types. Among these, tumors included multiple PTC variants or a combination of minimally invasive FTC and PTC (**Figure 1**).

The reasons for surgery in thyroid nodules classified as Bethesda I, II, and III at our center were mainly based on large nodule size (≥4 cm) or interval growth compared to previous measurements according to the ATA criteria, often accompanied by compressive symptoms or tracheal deviation. Surgery was also performed in a few cases based on patient preference large or increasing size, *n* = 73 (63.5%); patient preference, *n* = 17 (14.9%)]. Surgery was also performed when the FNA appeared benign but included an additional comment suggesting that follicular neoplasm or PTC could not be completely excluded or when the nodule was diagnosed as non-diagnostic or AUS on two or more repeated evaluations (indeterminate FNA results, *n* = 31, 27.0%). Additionally, some surgeries were performed for cosmetic reasons. In most cases, multiple reasons were involved (**Table 1**).

**Table 1:** Reasons for surgery in Bethesda class I and II thyroid nodules.

Reasons for surgery in Bethesda class I and II thyroid nodules	*N* = 115 (%)
Size above 4 cm or increased size	73 (63.5)
Cytology additional comment as “cannot be excluded follicular neoplasm or PTC”	25 (21.7)
Patient preference (aesthetic issue, medical condition, etc.)	17 (14.9)
Non-diagnostic result	6 (5.2)
USG features show suspicious malignancy	3 (2.6)

Some cases have surgery within several reasons and all were counted.

**Figure 2:**
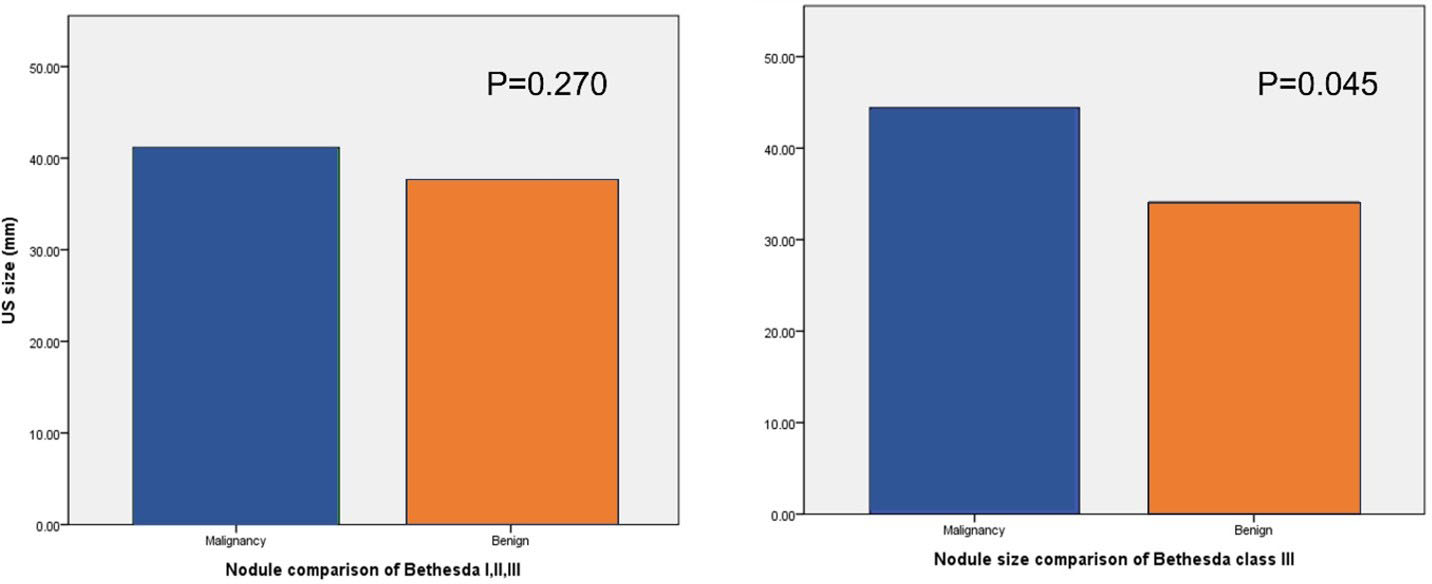
Comparison of the size of malignant and benign nodules confirmed after operation.

Nodules were classified as benign or malignant based on postoperative histopathologic results. The mean nodule size on preoperative USG was 3.73 ± 1.56 cm in the benign group and 4.09 ± 2.24 cm in the malignant group, with no statistically significant difference (*p* = 0.270) (**Figure 2**). In the univariate analysis, when comparing USG impression, presence of compressive symptoms, nodule size growth, and tracheal deviation, no significant statistical differences were observed (**Table 2**). However, Bethesda classification presented the significantly different malignancy rates (*p* < 0.001). Trachea deviation was likely to be related to the risk of malignancy (*p* = 0.053). In multivariable logistic regression (reference = Bethesda III), Bethesda II showed significantly lower odds of malignancy (OR: 0.28, 95% CI: 0.14–0.55, *p* < 0.001), whereas Bethesda I showed a non-significant decrease (OR: 0.38, 95% CI: 0.11–1.33, *p* = 0.131). Tracheal deviation demonstrated a borderline increase in odds (OR 2.07, 95% CI 0.95–4.51, *p* = 0.067) (**Table 3**).

**Table 2:** Univariate analysis of Bethesda class I, II and III thyroid nodule.

Parameters	Category	Malignancy (n = 62)	Benign (n = 130)	p-value
Sex, n(%)	Male	18 (29.0)	33 (25.4)	0.593
	Female	44 (71.0)	97 (74.6)	
Age, mean (range)		50.71	54.8	0.056
	Male	49.33 (21-77)	53.12 (33-71)	
	Female	51.27 (20-79)	55.40 (22-80)	
USG nodule size (cm)		4.09	3.73	0.270
USG impression, n(%)	Benign	2 (3.2)	5 (3.8)	0.301
	low	39 (62.9)	95 (73.1)	
	intermediate	15 (24.2)	25 (19.2)	
	high	6 (9.7)	5 (3.8)	
Bethesda Class, n(%)	I	4 (6.5)	13 (10.0)	**<0.001**
	II	20 (32.3)	78 (60.0)	
	III	38 (61.3)	39 (30.0)	
Symptom (globus dysphagia & dyspnea), n(%)	Yes	11 (17.7)	37 (28.5)	0.109
	No	51 (82.3)	93 (71.5)	
Growing, n(%)	Yes	18 (29.0)	41 (31.5)	0.725
	No	44 (71.0)	89 (68.5)	
Tracheal deviation, n(%)	Yes	18 (29.0)	22 (16.9)	0.053
	No	44 (71.0)	108 (83.1)	

Bold values indicate statistically significant p-values (p < 0.05).

**Table 3:** Multivariate analysis of the malignancy risk factors of Bethesda class I, II, III thyroid nodules.

Factor	OR	95% CI	P-value
Bethesda (1 vs 3)	0.38	0.11-1.33	0.131
Bethesda (2 vs 3)	0.28	0.14-0.55	**<0.001**
Trachea deviation	2.07	0.95-4.51	0.067
Age	0.98	0.95-1.00	0.054

Bold values indicate statistically significant p-values (p < 0.05).

Because Bethesda class was most significantly related to the risk of malignancy, a subgroup analysis was conducted for the patients with Bethesda class III nodules. The nodule size in the malignant group was slightly larger than that in the benign group (malignant: 4.39 ± 2.10 cm versus benign: 3.46 ± 1.44 cm, *p* = 0.045) (**Figure 2**). When atypia was divided into N/A atypia and others atypia types, the N/A atypia group showed a significantly higher malignancy rate (*p* = 0.028) in the univariate analysis (**Table 4**). A multivariate regression analysis was performed for Bethesda class III subgroup, and although the USG nodule size showed significance in the univariate analysis, no statistical significance was observed in the multivariate analysis (OR = 6.10, 95% CI: 0.994–37.451, *p* = 0.051). Significant results were found only for the cytology-related factors, with the N/A atypia showing approximately 13 times higher malignancy risk compared to the others atypia (odds ratio: 13.275, 95% CI: 1.354–130.2, *p* = 0.026) (**Table 5**).

**Table 4:** Subgroup univariate analysis of Bethesda class III thyroid nodule.

Parameters	Category	Malignancy (n = 38)	Benign (n = 39)	p-value
Sex n (%)	Male	14 (36.8)	10 (25.6)	0.289
	Female	24 (63.2)	29 (74.4)	
Age, mean (range)		48.84	54.79	0.063
	Male	48.21 (21-72)	54.80 (35-70)	
	Female	49.21 (20-79)	54.79 (22-80)	
USG nodule size (cm)		4.38	3.53	**0.045**
USG impression, n(%)	Low	26 (68.4)	26 (66.7)	0.914
	Intermediate	9 (23.7)	10 (25.6)	
	High	3 (7.9)	3 (7.7)	
Atypia, n(%)	Nuclear and architectural atypia	7 (18.4)	1 (2.6)	0.028
	Others	30 (78.9)	37 (94.9)	
Symptom (globus dysphagia & dyspnea) n (%)	Yes	7 (18.4)	13 (33.3)	0.136
	No	31 (81.6)	26 (66.7)	
Growing n (%)	Yes	10 (26.3)	11 (28.2)	0.852
	No	28 (73.7)	28 (71.8)	
Tracheal deviation n (%)	Yes	12 (31.6)	7 (17.9)	0.165
	No	26 (68.4)	32 (82.1)	

Bold values indicate statistically significant p-values (p < 0.05).

**Table 5:** Multivariate analysis of the malignancy risk factors of Bethesda class III thyroid nodules.

Factor	OR	95% CI	*P*-value
Sex	1.121	(0.351–3.576)	0.848
Age	1.033	(0.993–1.075)	0.105
Cytology (nuclear and architectural atypia)	13.275	(1.354–130.200)	**0.026**
Symptom (globus dysphagia and dyspnea)	0.375	(0.101–1.393)	0.143
Growing	1.515	(0.437–5.249)	0.512
Trachea deviation	1.319	(0.282–6.164)	0.725
USG impression (high versus low)	1.296	(0.180–9.333)	0.797
USG impression (intermediate versus low)	1.444	(0.229–9.107)	0.696
USG size	6.103	(0.994–37.451)	0.051

Statistically significant p-values are indicated in bold.

## Discussion

The current Bethesda class helps us to predict malignant potential and to guide treatment modality. Surgical resection for nodules with class I, II, and III is usually indicated when malignancy is suspected, such as in cases of progressive growth and signs of compression (5). Particularly in the case of AUS, surgery is considered when USG findings suggest malignancy (such as taller-than-wide shape, punctate echogenic foci, or irregular margins) or when molecular testing of BRAF mutation is detected (4). In this study, tracheal deviation, compressive symptoms, and Bethesda class III were related to the risk of malignancy. Bethesda class III was only significantly related to malignant risk. Considering that the subclassification of atypia in class III has currently emerged, we evaluated the significance of atypia in addition to previously known risk factors. Among the types of atypia, N/A atypia was a significant risk factor of malignancy.

Our study of the malignancy rates for Bethesda classes I, II, and III thyroid nodules were 23.5%, 20.4%, and 49.4%, respectively. In the case of AUS, previous literature reports an ROM ranging from 13% to 30%, but our study found a higher ROM of 49.4%, and differences in malignancy rates were also observed depending on the type of atypia (4). This higher ROM is likely to be influenced by referral bias, as we analyzed only the AUS cases that underwent surgery except surveillance cases. If the ROM were assessed for all AUS cases, it would likely be lower. Another possible reason is that our institution is a tertiary hospital, where many patients have already undergone sufficient follow-up or were referred for surgery due to the necessity of the procedure (11).

The primary reasons of our center for surgery on thyroid nodules classified as Bethesda classes I and II were the increase in nodule size and associated symptoms. However, when compared with surgical outcomes, nodule size and associated symptoms were found to be insignificant. This aligns with previous literature reviews and recent ATA guidelines (5, 6). Therefore, it is believed that surgery should not be considered based on any strict cutoff value of nodule size (6).

In the subgroup analysis within Bethesda class III, the univariate analysis revealed a difference in nodule size between malignant and benign groups, with the malignant group showing slightly larger nodules. However, this statistical significance was not confirmed in the multivariate analysis. Various studies have debated the correlation between nodule size and malignancy (12, 13). Caroline et al. reported that nodules larger than 4 cm in the AUS group have a higher risk of malignancy, while Hadi et al. suggested that nodules smaller than 2 cm are more likely to be malignant (12, 13). On the other hand, Dawish et al. concluded that there is no significant correlation between nodule size and malignancy and that size alone is not a reliable indicator of malignancy (14, 15).

In this study, a multivariate analysis was conducted not only on nodule size but also on sonographic impression, which included factors such as echogenicity, contents, margins, and suspicious malignancy features. A larger nodule size on USG was associated with an increased odds of malignancy. However, this did not reach statistical significance (OR: 6.103, 95% CI: 0.994–37.451, *P* = 0.051), showing a borderline trend. No other variables were significantly associated with malignancy in the multivariable analysis. While nodule size and sonographic impressions cannot be ignored in ultrasound evaluation, a more cautious interpretation is necessary when determining the management of Bethesda class III nodules (16, 17). Although the Youden index was maximized at a cutoff value of 5.26 cm, the corresponding sensitivity was only 31.6%, which was considered insufficient for clinical decision-making. Therefore, a lower cutoff value was selected to maintain a sensitivity above 60% while preserving reasonable specificity. A cutoff value of 3.55 was chosen, as it provided a balanced trade-off between sensitivity (63.2%) and specificity (53.8%).

The 2023 TBSRTC recommends that AUS cases should be further subclassified into two detailed categories: nuclear atypia and others (4, 18, 19). Nuclear atypia is characterized by mildly enlarged nuclei with slightly pale chromatin and limited nuclear contour irregularity (19). In “others” type, there are some subclasses: architectural atypia, oncocytic atypia, atypical lymphoid cell, and NOS. Architectural atypia is defined by the presence of microfollicles or crowded three-dimensional groups with scant colloid (19). Oncocytic atypia shows exclusively of oncocytic cells with minimal colloid. When atypical lymphocyte infiltration is observed but is not characteristic enough to be diagnosed as lymphoma, it is described as “atypical lymphoid cells.” (19) When atypia shows a minor population of follicular cells with nuclear enlargement and prominent nucleoli but has less concern of papillary carcinoma, then it is fit to classify such as NOS (19).

Within Bethesda class III, it is well known that nodules with nuclear atypia had a higher ROM compared to those without (4, 20, 21). Although this study did not show statistically significant results, the ROM was observed to be higher in cases with nuclear atypia. In this study, the Bethesda class III subgroup was analyzed by dividing cases into those showing both N/A atypia and those that did not. The results showed that nodules with both N/A types of atypia had a higher ROM than the others. The calculated odds ratio indicated an approximately 13-fold increased risk of malignancy in nodules with both N/A atypia. According to the 2023 TBSRTC, there is no difference in the ROM between nuclear atypia and N/A atypia. Therefore, both N/A atypia is included in the category of nuclear atypia. The discrepancy between our data and the existing guidelines seems to be primarily due to the fact that our dataset includes only samples from patients who underwent surgery. The reason that these patients underwent surgery is typically because malignancy could not be excluded, and as a result, the patients selected for this study may have characteristics that differ from those in the general population. This likely leads to a different interpretation of the data compared to the existing guidelines. Although there are inherent limitations in the data due to selection bias, it could still provide a stronger rationale for recommending surgery more proactively in patients with N/A atypia who may be hesitant about undergoing surgery.

Currently, we are utilizing repeated core needle biopsy (CNB) with or without BRAF testing, as well as diagnostic lobectomy, for nondiagnostic nodules. However, we still lack a reliable single diagnostic modality. Although CNB is often considered a more accurate diagnostic tool than FNA, the difference in diagnostic accuracy between the two methods seems to be minimal. In fact, previous studies have demonstrated a significantly higher conclusive rate for CNB compared to FNA only in specific cases, such as thyroid nodules larger than 2 cm and classified as ACR TIRAD or K-TIRADS category 4. Outside of these particular subgroups, the diagnostic yield of CNB and FNA does not differ significantly (22). In the present study, there were 22 cases meeting these criteria, among which nine cases were malignant. If CNB had been performed in these cases, a more accurate and refined preoperative diagnosis might have been achieved.

In this context, several molecular tests have been developed to improve the diagnostic accuracy of FNA cytology and to predict tumor behavior, potentially aiding preoperative surgical planning (10). Studies have demonstrated that high-risk molecular profiles are associated with more aggressive tumor characteristics, including larger tumor size, higher rates of nodal metastasis, vascular invasion, and shorter recurrence-free survival (10). However, despite these potential advantages, molecular testing is not routinely performed in all patients in real-world clinical practice. Therefore, clinical and cytopathologic findings remain the primary determinants of surgical decision-making in our study.

The overall prognosis for malignant cases of Bethesda classes I, II, and III nodules was excellent. Among the 62 patients of the malignant group, nodal recurrence was observed in only one case. The average follow-up period was 5.9 years, and no mortality was reported. The single case of nodal recurrence was identified 3 months after RAI treatment as a 5-mm nodal lesion on ultrasound. Due to its small size, the lesion was managed with active surveillance rather than additional surgical intervention, and the patient remained in a well-controlled state for over 5 years.

This study has several limitations. First, as a single-center study with a relatively small sample size, the statistical power of the subgroup analyses may be limited. Second, due to the retrospective nature of this study and the limited availability of molecular testing during the study period, molecular data were not consistently available. Given the growing role of molecular testing in the risk stratification of indeterminate thyroid nodules, the absence of such data limits a more comprehensive evaluation of malignancy risk factors. Future prospective studies incorporating molecular testing are warranted to further validate and refine the risk stratification of indeterminate thyroid nodules. To assess diagnostic accuracy, all non-diagnostic nodules that underwent surgical resection were included in the analysis. However, because only surgically treated cases were analyzed, selection bias is inevitable. This may have contributed to the relatively higher observed malignancy rate, particularly in Bethesda category III nodules. Despite this limitation, our findings suggest that specific cytologic subcategories within Bethesda class III may be associated with a higher risk of malignancy and could serve as an additional predictive factor in clinical decision-making.

In conclusion, while controversies remain regarding the significance of the Bethesda class III group, our findings suggest that the presence of both N/A atypia is associated with a higher likelihood of malignancy. Nodule size measured by ultrasonography was not statistically significant in predicting malignancy; however, malignant nodules tended to be larger than benign ones in Bethesda class III. When considered alongside previously reported risk factors, the identification of both N/A atypia on preoperative cytopathology may support a more proactive surgical approach in selected patients. Pathophysiological relationship between atypia and malignant potential should be elucidated in a future study.

## Data Availability

The datasets generated and/or analyzed during the current study are available from the corresponding author upon reasonable request and in accordance with institutional and ethical regulations.
